# Coevolutionary interactions between farmers and mafia induce host acceptance of avian brood parasites

**DOI:** 10.1098/rsos.160036

**Published:** 2016-05-04

**Authors:** Maria Abou Chakra, Christian Hilbe, Arne Traulsen

**Affiliations:** 1 Department of Evolutionary Theory, Max Planck Institute for Evolutionary Biology, August-Thienemann-Straße 2, 24306 Plön, Germany; 2 Program for Evolutionary Dynamics, Harvard University, Cambridge, MA 02138, USA; 3 IST Austria, Am Campus 1, 3400 Klosterneuburg, Austria

**Keywords:** evolutionary game theory, coevolution, retaliation, punishment, host–parasite interactions

## Abstract

Brood parasites exploit their host in order to increase their own fitness. Typically, this results in an arms race between parasite trickery and host defence. Thus, it is puzzling to observe hosts that accept parasitism without any resistance. The ‘mafia’ hypothesis suggests that these hosts accept parasitism to avoid retaliation. Retaliation has been shown to evolve when the hosts condition their response to mafia parasites, who use depredation as a targeted response to rejection. However, it is unclear if acceptance would also emerge when ‘farming’ parasites are present in the population. Farming parasites use depredation to synchronize the timing with the host, destroying mature clutches to force the host to re-nest. Herein, we develop an evolutionary model to analyse the interaction between depredatory parasites and their hosts. We show that coevolutionary cycles between farmers and mafia can still induce host acceptance of brood parasites. However, this equilibrium is unstable and in the long-run the dynamics of this host–parasite interaction exhibits strong oscillations: when farmers are the majority, accepters conditional to mafia (the host will reject first and only accept after retaliation by the parasite) have a higher fitness than unconditional accepters (the host always accepts parasitism). This leads to an increase in mafia parasites’ fitness and in turn induce an optimal environment for accepter hosts.

## 1. Introduction

Interactions between species, whether antagonistic, competitive or mutualistic, can drive evolution and diversification. Coevolution between antagonistic species involves constant adaptation and counter adaptation. A fascinating example is brood parasitism, where the parasites avoid the costs of parental care by laying their eggs in a host’s nest. This elicits coevolutionary responses: hosts acquire defences to escape from parasitism, and the parasites in turn develop new ways to trick the host. There are numerous ways a host can resist parasitism; for example, by using egg signatures to identify the foreign egg, or by synchronizing nesting to limit parasitism occurrences [1–4]. Thus, it remains puzzling why some hosts accept any form of parasitism [5,6].

To address this question, several studies have modelled these host–parasite interactions (i.e. [7–12]), including models that explicitly focus on the ‘mafia hypothesis’ [1,13–15]. According to these models, hosts should accept parasitism when it is likely that a parasite would retaliate [8–10]. Simulations showed that the respective models can lead to cyclic patterns where retaliation evolves when host rejection is abundant [8–10]. Retaliatory behaviour, in turn, can emerge when hosts have evolved plastic behaviours, and when hosts are likely to reject the first parasitism attempt [11]. This is in line with experimental evidence showing that retaliation can lead hosts to accept brood parasites in subsequent nests [14,15]. On the population level, these adaptations and counter-adaptations cause coevolutionary cycles: when retaliatory behaviour (the so-called ‘mafia’) is abundant, there is a fitness advantage for accepter hosts. However, as the frequency of accepter hosts increases, it is no longer necessary for parasites to retaliate, and non-retaliators increase in frequency. Once the threat of retaliation has waned, hosts should reject first parasitism attempts, selecting for retaliatory behaviour again and thereby closing the evolutionary cycle. These models provide a glimpse into the complex frequency-dependent effects of parasitism. The resulting coevolutionary arms race leads to oscillations with one species lagging behind the other [8–11].

Although experimental evidence shows that avian brood parasites, such as the great spotted cuckoos or the brown-headed cowbirds, exhibit retaliatory behaviour [14,15], the mafia hypothesis is still controversial [16,17]. Retaliatory behaviour seems too costly to emerge in the first place. Mafia parasites would need to observe the respective host’s nest and after recognizing that their egg was rejected, they would need to punish the host by destroying the entire nest. Instead, it has been suggested that destroyed nests are not a consequence of retaliatory behaviour, but of farming behaviour [1,14,15,18]. Farming parasites use depredation to synchronize the timing with the host. They destroy mature clutches to force the host to re-nest, which creates a new opportunity for the parasite. Thus, both farming and mafia behaviour involve depredation as a tactic, but only mafia parasites use depredation as a targeted response to rejection. However, it remains difficult to empirically distinguish retaliatory behaviour from farming behaviour [1,14,15,18], thus making it hard to disentangle whether host acceptance is driven by depredation or retaliation.

A recent theoretical study incorporated both depredatory behaviours into a single model, and found that both types of depredators are favoured over non-depredators when hosts show a plastic response [19]. Surprisingly, however, the model also suggested that hosts should not exhibit any plastic response in the first place. Instead, hosts should always reject parasitic eggs, independent of any former interactions. This conclusion contradicts previous work proposing that host acceptance should naturally evolve when retaliation is common [8–11]. The model in [19] assumes that after depredation, retaliatory parasites are not more likely to return to the same host than are non-retaliatory parasites. However, for a mafia strategy to be successful, it seems to be critical that retaliatory parasites revisit the same host—in fact, previous simulations have confirmed that retaliators can gain a higher fitness from returning [11]. When mafia parasites are likely to return, there is also a strong evolutionary pressure for hosts to accept parasitism. This simulation result has also been observed experimentally [15]—warblers (hosts) that accepted the parasite produced significantly more offspring than the ones that rejected the parasite. Experimental evidence also shows that retaliation may not only promote acceptance, but it may also delay the evolution of rejector hosts [15,18,20]. These arguments indicate that host acceptance may be a robust strategy, even when we include farming as a possible behaviour of the parasite. Herein, we aim to explore this question in detail. We use an evolutionary model to describe the interaction of farmers and mafia parasites, and we analyse how farming changes the way hosts respond to brood parasitism.

## 2. Model

**Figure 1.**
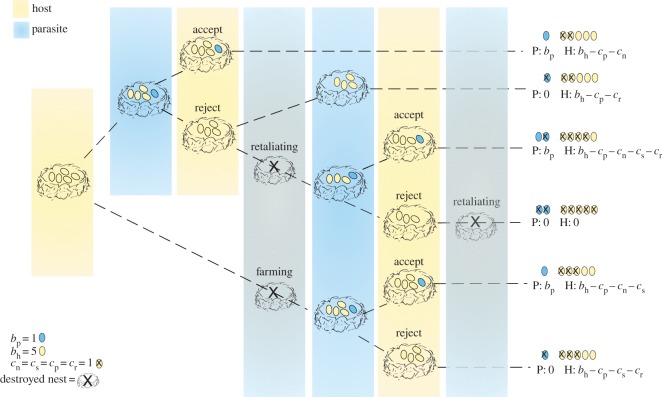
Game-tree for the host–parasite interaction. The host lays *b*_h_ eggs in a clutch, which may become parasitized (at a cost *c*_p_) or depredated. Depredation forces the host to re-nest, costing *c*_s_. Hosts may reject (at cost *c*_r_) or accept the parasitic egg and incur nestling cost *c*_n_. The parasite gains the accepted egg.

Based on [11], we have developed a model that incorporates both farming and mafia parasites. The host–parasite interaction takes the form of a game (figure 1), in which the breading season is divided into several stages. Initially, the host lays *b*_*h*_ eggs in a clutch that can either be parasitized or depredated by a parasite. After parasitism, the host may accept the parasitic egg incurring a parasitism cost *c*_*p*_ and a nestling cost *c*_*n*_. Alternatively, the host may reject the parasitic egg at a cost *c*_*r*_ (the rejection cost encompasses a host’s ability to recognize and eject the foreign egg) and risk retaliation. If the nest is depredated, either by a farmer (before parasite eggs are laid) or by a retaliator (after her egg was rejected), hosts are forced to re-nest at a cost *c*_*s*_.

For the evolutionary model, we consider four types of hosts behaviours: two unconditional behaviours, which are to always accept or reject parasitism, and two conditional behaviours, which are to accept only after depredation or only after retaliation. The conditional behaviours mimic a host’s ability to learn and change as a response to repeated interactions with the parasite [21]. For the parasites, we consider three types of behaviour: (i) non-depredating parasites; (ii) mafia retaliators that depredate the nest after rejection. They re-parasitize the same host, and they destroy all future clutches unless acceptance occurs; and (iii) farmers depredate the host’s nest in the first stage to create an opportunity for parasitism. For simplicity, we neglect the possibility of parasites that engage in both farming and retaliation simultaneously—in particular, farmers do not return to the host after parasitism.

The fitness of each type is calculated at the end of a season. Non-depredatory parasites *N* lay a total of *β*_N_ eggs per season, whereas mafia and farming parasites, *M* and *F*, lay a total of *β*_M_ and *β*_F_ eggs, respectively. We assume *β*_M_≤*β*_F_ and *β*_M_≤*β*_N_ to incorporate that depredation may be costly for the mafia parasite (for example, because of cognitive and energy costs, [16]). We do not presume any particular relationship between between *β*_F_ and *β*_N_. The case *β*_F_<*β*_N_ may be justified because farming requires more efforts than non-depredating behaviour. On the other hand, *β*_F_≥*β*_N_ may be justified because farmers are able to create additional parasitism opportunities. The three types of parasites have frequencies denoted by *x*_N_, *x*_M_ and *x*_F_, such that *x*_N_+*x*_M_+*x*_F_=1. Likewise, the four types of hosts, accepters *A*, conditional to mafia CM, conditional to depredation CD, and rejecters *R* have respective frequencies *y*_A_, *y*_*CM*_, *y*_CD_ and *y*_R_ such that *y*_A_+*y*_CM_+*y*_CD_+*y*_R_=1.

We calculate the average fitness of each parasite by considering the average number of accepted eggs reared by the host. To this end, we also need to consider the average number of laid eggs per host interaction. Non-depredatory parasites lay a single egg per parasitized host nest, which only becomes accepted if the host happens to be an accepter. For a mafia parasite, the average number of eggs laid per parasitized host nest is *y*_A_+2*y*_CM_+2*y*_CD_+2*y*_R_=2−*y*_A_, whereas the expected number of accepted eggs per parasitized host nest is *y*_A_+*y*_CM_+*y*_CD_. Finally, farmer parasites lay a single egg per nest, and the expected number of accepted eggs per parasitized host is *y*_A_+*y*_CD_. The average fitness of a parasite is thus given by

2.1 $$\begin{array}{rl} \pi_{N} & =y_{A}\cdot \beta_{N},\\ \pi_{M} & =\frac{y_{A}+y_{\mathrm{CM}}+y_{\mathrm{CD}}}{2-y_{A}}\cdot \beta_{M},\\ \pi_{F} & =(y_{A}+y_{\mathrm{CD}})\cdot \beta_{F}. \end{array}\}$$

To derive the average fitness of the hosts, we first need to calculate the probability with which a host is visited by each parasite type. Farmers and non-depredatory parasites visit *β*_F_ and *β*_N_ different hosts, respectively, whereas mafia parasites require on average *β*_M_/(2−*y*_A_) different hosts. Thus, the probability that a foreign egg in a host’s nest comes from a non-depredatory parasite, farmer, or mafia parasite is proportional to *x*_N_*β*_N_, *x*_F_*β*_F_ or *x*_M_*β*_M_/(2−*y*_A_), respectively. As a consequence, the average fitness of a parasitized host becomes

2.2 $$\begin{array}{rl} \pi_{A} & =b_{h}-c_{p}-c_{n}-\frac{c_{s}\cdot \beta_{F}\cdot x_{F}}{\beta_{F}x_{F}+\beta_{N}x_{N}+\beta_{M}x_{M}/(2-y_{A})},\\ \pi_{\mathrm{CM}} & =b_{h}-c_{p}-c_{r}-\frac{(c_{n}+c_{s})\cdot \beta_{M}\cdot x_{M}/(2-y_{A})+c_{s}\cdot \beta_{F}\cdot x_{F}}{\beta_{F}x_{F}+\beta_{N}x_{N}+\beta_{M}x_{M}/(2-y_{A})},\\ \pi_{\mathrm{CD}} & =b_{h}-c_{p}-\frac{(c_{s}+c_{n}+c_{r})\beta_{M}x_{M}/(2-y_{A})+c_{r}\beta_{N}x_{N}+(c_{s}+c_{n})\beta_{F}x_{F}}{\beta_{F}x_{F}+\beta_{N}x_{N}+\beta_{M}x_{M}/(2-y_{A})},\\ \pi_{R} & =\frac{(b_{h}-c_{p}-c_{r})\cdot \beta_{N}x_{N}+(b_{h}-c_{p}-c_{s}-c_{r})\cdot \beta_{F}\cdot x_{F}}{\beta_{F}x_{F}+\beta_{N}x_{N}+\beta_{M}\cdot x_{M}/(2-y_{A})}. \end{array}\}$$

To model the population dynamics, we assume that the frequencies of each type change according to the replicator equation, ${\dot{x}}_{i}=x_{i}(\pi_{i}-\bar{\pi })$ for the parasites with $\bar{\pi }=\sum_{j}\pi_{j}x_{j}$ and ${\dot{y}}_{i}=y_{i}(\pi_{i}-\bar{\pi })$ for the hosts with $\bar{\pi }=\sum_{j}\pi_{j}y_{j}$ [22]. That is, strategies that yield a fitness above the population average are expected to spread, whereas strategies that yield a fitness below the population average decrease over time.

## 3. Results and discussion

**Figure 2.**
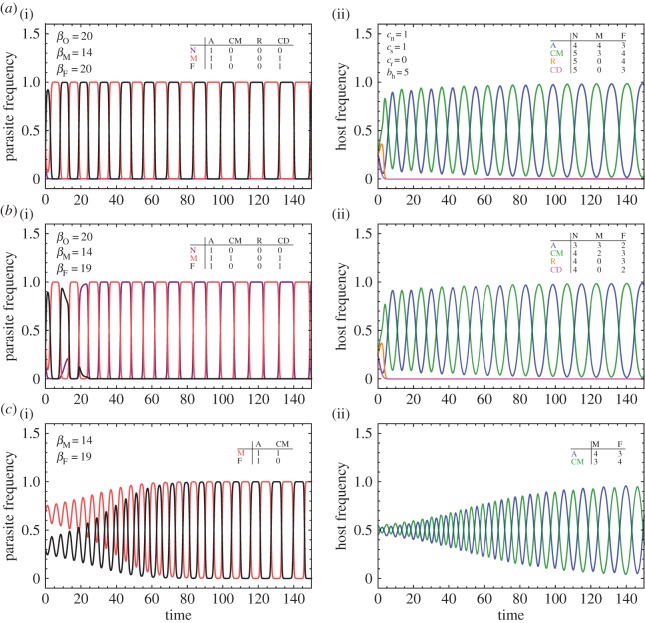
Dynamics of host–parasite interactions. The figure shows frequencies of parasites (*a*(i),*b*(i),*c*(i)) and hosts (*a*(ii),*b*(ii),*c*(ii)). (*a*) When *β*_F_=*β*_N_, we find oscillations between mafia and farmer parasites, and between conditional accepter and accepter hosts. (*b*) When *β*_F_<*β*_N_, the non-predatory parasites displace the farmers, leading to oscillations between mafia and non-depredatory parasites, and between conditional to mafia and unconditional accepter hosts. (*c*) For a simplified model with only two strategies present in each population, we can show the existence of a mixed equilibrium. However, that equilibrium is unstable; nearby initial populations follow cycles with increasing amplitude. The insets shows the fitness table arising from a single host–parasite interaction: however, owing to the nonlinear interaction, this cannot be directly interpreted as a game-theoretical pay-off matrix, see equations (2.1) and (2.2).

We fist explore the strategy dynamics of two different situations, one which assumes *β*_M_<*β*_F_=*β*_N_ and the other *β*_M_<*β*_F_<*β*_N_, as shown in figure 2. When farmers lay at least as many eggs as non-depredating parasites, we observe coevolutionary cycles between mafia and farmer parasites, and between accepter hosts (always accepting) and conditional accepter hosts (reject first and accept after retaliation). Conversely, when farmers have a disadvantage compared with the *N* type, we observe coevolutionary cycles between non-depredatory parasites and mafia parasites, and between accepter hosts and conditional accepter hosts. Both examples illustrate that accepter hosts can have a temporary fitness advantage over other host types.

These evolutionary outcomes can be understood explicitly. First, unconditional rejection is never optimal because conditional behaviour leads to a higher fitness, *π*_CM_≥*π*_R_. Similarly, given that *c*_*r*_<*c*_*n*_, it is better for hosts to condition their behaviours on retaliation rather than on depredation, as *π*_CM_≥*π*_CD_. Hence, the abundance of CD is expected to drop in any mixed population, and eventually *y*_CD_≈0. Furthermore, the laid eggs per season determine whether farmers or non-depredating parasites gain more offspring. When *β*_F_≥*β*_N_, farming is more profitable, *π*_F_≥*π*_N_, and we can simplify our model to a parasite population composed of just depredators (mafia and farmers) and a host population composed of unconditional accepters and accepters conditional to mafia. For this reduced system, the replicator dynamics simplifies to

3.1 $$\begin{array}{rl} {\dot{x}}_{M} & =x_{M}(1-x_{M})(\pi_{M}-\pi_{F}),\\ {\dot{y}}_{A} & =y_{A}(1-y_{A})(\pi_{A}-\pi_{\mathrm{CM}}). \end{array}\}$$

Where the remaining abundances are *x*_F_=1−*x*_M_ and *y*_CM_=1−*y*_A_. For *c*_*r*_>*c*_*n*_, the dynamics has no interior equilibrium and mafia behaviour will vanish. For *c*_*r*_<*c*_*n*_, the dynamics has an equilibrium $\left(x_{M}^{*},y_{A}^{*}\right)$ in the interior of the state space, which is from *π*_M_=*π*_F_ and *π*_A_=*π*_CM_,

3.2 $$\begin{array}{rl} x_{M}^{*} & ={\left(1+\frac{c_{s}+c_{r}}{c_{n}-c_{r}}\left(1-\sqrt{1-\frac{\beta_{M}}{\beta_{F}}}\right)\right)}^{-1},\\ y_{A}^{*} & =1-\sqrt{1-\frac{\beta_{M}}{\beta_{F}}}. \end{array}\}$$

However, this equilibrium is unstable (appendix A), and in the long-run the dynamics of this host–parasite interaction exhibits strong oscillations (as depicted in figure 2). If most parasites engage in farming, hosts that condition acceptance to just after retaliation have a higher fitness than unconditional accepters. As the frequency of conditional accepters increases, the mafia parasites’ fitness supersedes the fitness of farmers. In turn, as mafia parasites increase in frequency, they induce an optimal environment for unconditional accepter hosts, eventually leading the parasite population back to the farmer strategy.

Interestingly, one can observe exactly the same dynamics when exploring the competition between non-depredating parasites and mafia parasites [11]. Intuitively, for explaining the evolution of acceptance among hosts, the distinction between farming behaviour and non-depredating parasites becomes irrelevant. Both types evoke the same response among hosts, making it optimal for hosts to reject first attempts. Thus, our model suggests that if accepting behaviour is observed in a host population, it needs to be owing to the presence of retaliating parasites.

**Figure 3.**
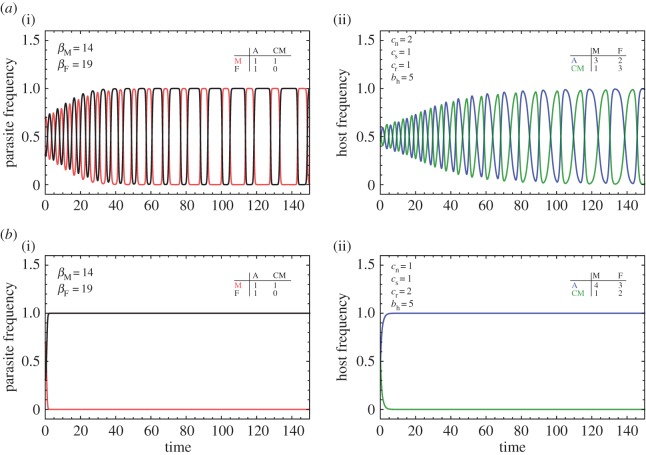
Exploring the effects of cost on host–parasite interactions when *β*_F_>*β*_*m*_. There are two cases that can alter the dynamics of the system: (*a*) when *c*_r_<*c*_n_, shows oscillations between mafia and farmers, and between conditional to mafia and accepter hosts. (*b*) When *c*_r_≥*c*_n_, the rejection cost inhibits the emergence of conditional accepters and thus only farmers benefit, as a result we have a host population composed of only accepters. The insets shows the fitness table arising from a single host–parasite interaction.

So far we have been able to show that even when both farmers and mafia interact, hosts will evolve to accept some degree of parasitism. While our theory predicts cyclical dynamics, it is still debated whether host–parasite interactions display any cycles at all [1,4,23,24]. In our model, there must be at least two types of hosts and parasites available for cycles to occur, which in turn depends on the costs incurred when hosts and parasites interact. So far we have assumed that the costs of raising a nestling *c*_n_ outweigh the rejection cost *c*_r_ (i.e. the cost for the host’s ability to recognize and eject a foreign egg without errors). To address whether accepter hosts are able to evolve when rejection costs are low, our numerical examples so far have assumed that the host incurs no extra costs when rejecting a foreign egg. Once we relax this assumption and consider the case *c*_r_>0, we identified that cycles tend to only occur when rejection costs are less than nestling costs, *c*_r_<*c*_n_ (see figure 3*a*, for an example with *c*_r_>0). In that case, one can show from the fitness equations in (2.2) that for hosts it is optimal always to accept parasitism, as *π*_A_≥*π*_CM_, *π*_A_≥*π*_CD_ and *π*_A_≥*π*_R_. The dynamics leads to a steady state in which hosts accept, and in which the parasite with the highest number of eggs *β*_*i*_ succeeds such as the example shown in figure 3*b*.

These regimes resemble the two competing hypothesis put forward to explain the lack of consensus seen experimentally: the first is the evolutionary lag hypothesis, which states that hosts would naturally evolve to defend against the current parasites which will then bring about change for the parasite who must evolve to survive. The problem is time—we may not catch the next step of the arms race, because there is a lag in their response and insufficient amount of time has passed for the adaptation to occur. The second is the equilibrium hypothesis, which states that what we observe is a stable state where hosts are behaving as they should [3,4]. Thus, our model suggests that if we assume low rejection costs relative to nestling costs, cycles will exist leading to an evolutionary lag (experimentally, it can be shown that raising a parasite’s egg incurs fitness loss for many hosts [2,4,23]). This prediction is in line with experimental results of a system where the rejection costs of a host are zero [24]. However, once rejection costs exceed nestling costs, the outcome will depend on the overall costs and competition within species. Future empirical work could aim to disentangle the costs of the hosts, and generalize the patterns observed in the model to the natural systems.

The general dynamical pattern discussed herein is consistent for both the simplified and complex model. However, these models proposed still involve a number of simplifications. In particular, we did not explicitly keep track of the time within a season (which is certainly an important variable to model the effects of farming). Instead, the advantages of farming were incorporated indirectly, by giving farmers additional opportunities to parasitize hosts. However, we expect our basic insights to be robust also for models that include more detail: depending on environmental conditions, farming may be a profitable strategy for brood parasites, but it does not induce hosts to accept parasitism. By contrast, acceptance can readily evolve when parasites engage in retaliatory behaviour, and if depredation is specifically targeted at hosts who have previously rejected the parasite’s eggs. Our model also highlights that there may be no optimal behaviour in these multispecies systems. Instead, hosts and parasites will typically remain in a recurring process of mutual adaptation.

## Appendix A

To explore whether the interior fixed point of the simplified model is stable, we linearized the system equation (3.1) around the fixed point (*x**_M_,*y**_A_). The corresponding Jacobian matrix takes the form

A 1 $$J=\left(\begin{array}{cc} 0 & a_{12}\\ a_{21} & a_{22} \end{array}\right).$$

The non-vanishing entries of *J* are

A 2*a* $$\begin{array}{rl} a_{12} & =\frac{-2\beta_{M}(c_{n}-c_{r})(c_{r}+c_{s})\sqrt{1-\beta_{M}/\beta_{F}}}{{\left((c_{r}+c_{s})(\beta_{M}/\beta_{F})+(c_{n}-c_{r})(1+\sqrt{1-\beta_{M}/\beta_{F}})\right)}^{2}}, \end{array}$$

A 2*b* $$\begin{array}{rl} a_{21} & =\frac{\sqrt{1-\beta_{M}/\beta_{F}}\cdot {\left((c_{n}-c_{r})+(c_{r}+c_{s})(1-\sqrt{1-\beta_{M}/\beta_{F}})\right)}^{2}}{c_{n}+c_{s}}, \end{array}$$

A 2*c* $$\begin{array}{rl} a_{22} & =\frac{\left(c_{n}-c_{r})(c_{r}+c_{s}\right)}{c_{n}+c_{s}}\left(2-\sqrt{1-\frac{\beta_{M}}{\beta_{F}}}-2\frac{\beta_{F}}{\beta_{M}}\left(1-\sqrt{1-\frac{\beta_{M}}{\beta_{F}}}\right)\right). \end{array}$$

Under the assumption that *β*_M_<*β*_F_ and *c*_r_<*c*_n_, we have *a*_12_<0 and *a*_21_>0. In addition, we note that *a*_22_>0 because the function $f(z)=2-\sqrt{1-z}-(2/z)(1-\sqrt{1-z})$ is strictly positive in the interval *z*∈(0,1). The Eigenvalues of the Jacobian matrix can be written as $\lambda_{1,2}=((\text{tr}\;J)\pm \sqrt{{\left(\text{tr}\;J\right)}^{2}-4(\det J)})/2$, where tr *J*=*a*_22_>0 is the trace and $\det J=-a_{12}a_{21}>0$ is the determinant of the Jacobian matrix. As (tr *J*)>0, the interior fixed point is unstable. Since none of the boundary fixed points is stable (each of the pure host or parasite strategies can be invaded), the Poincaré–Bendixson theorem (e.g. [25]) implies that the coevolutionary dynamics exhibits oscillations.
